# Towards accurate models for predicting smartphone applications’ QoE with data from a living lab study

**DOI:** 10.1007/s41233-020-00039-w

**Published:** 2020-10-04

**Authors:** Alexandre De Masi, Katarzyna Wac

**Affiliations:** 1 University of Geneva, Geneva, Switzerland; 2 University of Copenhagen, Copenhagen, Denmark

**Keywords:** Quality of experience, Context, Quality of service, Mobile applications expectation, Machine learning

## Abstract

Progressively, smartphones have become the pocket Swiss army knife for everyone. They support their users needs to accomplish tasks in numerous contexts. However, the applications executing those tasks are regularly not performing as they should, and the user-perceived experience is altered. In this paper, we present our approach to model and predict the Quality of Experience (QoE) of mobile applications used over WiFi or cellular network. We aimed to create predictive QoE models and to derive recommendations for mobile application developers to create QoE aware applications. Previous works on smartphone applications’ QoE prediction only focus on qualitative or quantitative data. We collected both qualitative and quantitative data “in the wild“ through our living lab. We ran a 4-week-long study with 38 Android phone users. We focused on frequently used and highly interactive applications. The participants rated their mobile applications’ expectation and QoE and in various contexts resulting in a total of 6086 ratings. Simultaneously, our smartphone logger (mQoL-Log) collected background information such as network information, user physical activity, battery statistics, and more. We apply various data aggregation approaches and features selection processes to train multiple predictive QoE models. We obtain better model performances using ratings acquired within 14.85 minutes after the application usage. Additionally, we boost our models’ performance with the users expectation as a new feature. We create an on-device prediction model with on-smartphone only features. We compare its performance metrics against the previous model. The on-device model performs below the full features models. Surprisingly, among the following top three features: the intended task to accomplish with the app, application’s name (e.g., WhatsApp, Spotify), and network Quality of Service (QoS), the user physical activity is the most important feature (e.g., if walking). Finally, we share our recommendations with the application developers, and we discuss the implications of QoE and expectations in mobile application design.

## Introduction

Smartphone applications are used all the time in various contexts since their introduction. The majority of them depend on an Internet connection and other factors, linked to the smartphone hardware and software (e.g., processor, memory, video buffer) to become an enjoyable experience for the users. Quality of Experience (QoE) is defined by [1] as “the degree of delight or annoyance of the user of an application or a service”. QoE is profoundly shaped by the user expectations [2], previous experiences (i.e., expectations), and Quality of Service (QoS), (e.g., network speed), as well as other user contexts (e.g., mobility level). Collecting samples of QoE rating has always been difficult due to potentially confounding factors, including the user’s immediate context. The majority of the QoE studies are done in-lab or in-situ via crowdsourcing, where participants have to execute tasks given by the researchers and later rate their experience. The previous work focused on repetitive actions orchestrated by the study authors, executed by the study participant. Once finished, the action experience was rated and collected.

In our work, we focus on collecting QoE and expectation ratings from living lab participants unobtrusively in their daily life contexts. We propose a method to build smartphone applications’ QoE prediction models with data collected discreetly through a smartphone logger (mQoL-Log) and assisted by the Ecological Momentary Assessment (EMA) methodology “in the wild”. We focus on both qualitative and quantitative research for modeling QoE. This hybrid method added contextual information to design a QoE prediction model. This approach enables us to make recommendations to smartphone application developers. We train multiple prediction models to predict “High” or “Low” QoE. We perform extensive data wrangling and cleaning to build better predicting models with higher performance than our previous work [3]. We investigate different machine learning algorithms, as previously shown to be used in the literature [4].

Through our work, we present a path toward building an accurate QoE model from a dataset obtained in-the-wild. Contrary to an in-the-lab study, where researchers have full control over the stimulus applied to their participants. In-the-wild studies create new challenges, e.g., external factors influence the experiment. They have to be leveraged to extract knowledge from the collected data.

Current smartphone sensing technologies and the recent development in machine learning tools enable our path toward predictive QoE models executed directly on the smartphone. In the future, a smartphone should be able to anticipate the change in its QoE and clearly notify its user about the possible disappointment, (via, e.g., notification or even a screen color change).

The process of building QoE predictive models requires filtering and aggregating the raw data produced by the participant. We review the quality of our rated applications’ QoE samples and the performances of our aggregation solutions. We focus on the time between the application’s usage event and its rating. We explore the unlabeled tasks in the application and look into their usability to create an improved model. We investigate the impact of expectation on QoE and its correlation. We review the possibility of an on-device prediction model and its shortcoming. We investigate the most predictive features from our models to derive what factors affect smartphone applications’ QoE “in the wild”. Once established, we create a model to predict QoE based on these factors. To test the importance of various features for the QoE model, the paper describes an iterative model building methodology (i.e., including data filtering and wrangling techniques, as well as model evaluation metrics).

Modeling QoE “in the wild” requires data collection from various perspectives. mQoL-Log allows for context monitoring on Android smartphones. It enables extensive data collection of critical external factors linked to the participant annotation and the observed experiences. Smartphone-based context monitoring can become an issue if the privacy and experience of the participants are impacted. Hence, we design a study protocol to reduce the participant’s burden. If the data collection endeavour has not been carefully planned, the study can directly impact the user’s day to day smartphone-based activities and experiences.

This paper is structured as follows: the related work is in “Related work”. “The approach: user study” section introduces our study, its protocol, and lists the data collected. “Building QoE Prediction Models” section exposes the methodology of our work, the construction of our QoE prediction models, and shows its output. “Discussion” section discuses the models, its results, and our findings. “Study limitations” section presents the limitation of our study and “Conclusions and future work areas” section concludes the paper. In this paper, the term “accurate” refers to the QoE models’ performance (high accuracy: value close to 1, low: value close to 0), as used by the machine learning and QoE community.

## Related work

We focused our literature review on three main categories of papers. Firstly we looked at past QoE studies with mobile Internet devices. At that time, the most used mobile Internet devices were laptops connected to broadband Internet. Secondly, we investigated the works of quantifying QoE on smartphones. We explored the works of applications’ QoE on smartphones from framework to model. In our last category, we reviewed the works linking expectation and QoE, especially for smartphone as a platform.

### Quantifying QoE on laptop

In 2011 [5] Schatz et al. assessed mobile broadband quality in-situ and in-lab on laptops. The authors asked the participant to browse websites and to download files, then rate their experience. The network traffic was transferred from the broadband operator to the authors’ network shaper. Their following work the same year [6] mapped QoE ratings and the acceptability of a web service. The previously cited works were all done with instruction to rate tasks experience arranged by the authors. Their study setup modified the typical experience of their participants. Casas et al. [7] focused their work on the specific web services’ QoE in 2012. In their study, they collected QoE rating’s from Facebook browsing and Youtube usage “in the wild”. Their 33 participants used a laptop with a 3.5G mobile broadband connection provided by the authors for 31 days. The traffic was rerouted to the authors’ network before accessing the Internet. They applied traffic shaping to influence the participants’ QoE. The participants rated the quality of the connection and the overall experience on a MOS scale, as well as the acceptability of the service. They focused their approach on QoS metrics (e.g., downlink bandwidth, traffic volume, video resolution) to compute MOS score expressing QoE. They did not collect any context information except the physical location of the participant (home, work, university, outdoor and other) manually reported. Sackl et al. [8] in 2015 focused on Web QoE ratings of a photo gallery website and on overall quality for three uses: browsing a news website, uploading a large file and exploring different cities in Google Maps in a laboratory setting. They modulated the bandwidth and its stability to observe the participants quality perception.

### Smartphone applications’ QoE

DeMoor et al. [9] in 2010 created a framework to quantify mobile QoE for all smartphone applications in a living lab setting. They evaluated the QoE of Java platform applications in the implementation of their framework. However, they did not take into account the evolution in the cellphone landscape to smartphone and its generated services. The mobile Java platform was getting obsolete at that time (2010). The first Apple smartphone (iPhone) was released in 2007; the first Android device (HTC Dream) was available in 2008. They advocated for long-term and user-centric perspective QoE studies in living labs, without operationalizing it. The factors influencing mobile application QoE are various, as shown by Ickin et al. [10] in 2012 with their study assessing users’ perceived experience “in the wild”. The ratings were provided at random times after any application usage. The authors presented factors that impacted the users’ QoE, as the application interface design, the performance, the battery efficiency, the in-application features and the application name. They also exposed user-centred ones as connectivity cost, user routines, and user lifestyle. Other attempts have been made for quantifying QoE on Android OS smartphone devices. Chen et al. [11] in 2014 proposed a tool to monitor multiple QoE factors, including a QoE-aware User Interface (UI) controller injected in the Android OS application, the overall network QoS of the device, and the 4G/LTE modem state obtained by a cellular network diagnosis tool from the modem chip maker. In the case of video streaming on smartphones, Wamser et al. [12] in 2015 developed its Android OS application to collect QoE ratings of Youtube videos. The participants were invited to their lab to use specific smartphones connected to a WiFi network for which the authors adjusted the network QoS, notably its available bandwidth. The first study on QoE of mobile applications in real cellular networks was done by Casas et al. [13, 14] in 2015. They later combined in-lab study results from [15] and QoE ratings from a study “in the wild” about various mobile services [16]. They focused mainly on QoS and the annotation of their participants to derive bandwidth thresholds for good/bad QoE on cellular networks. However, their participants were instructed to perform specific tasks (e.g., watch a video on Youtube and explore a map on Google Maps), possibly interfering with their normal smartphone usage and creating a bias. They rated the QoE of that specific task in the authors’ application. It collected network flows information, e.g., Radio Access Technology (RAT). The flow metrics are not available anymore without root access on the Android OS. Google removed those API as a security concern in 2016. The dataset from this study was used later by Casas et al. [17] in 2017 to predict QoE comparing different machine learning classifiers. Decision Tree-based classifiers were proven to get the best results. The authors followed with [4, 18] to predict QoE with the benefit of ensemble models via their stacking approach [19]. In all their work, they did not integrate physical activity, user habits (e.g., time spent in the application), expectation or active network testing.

**Table 1:** Study EMAs questions

Questions	Answer	Type	Features
Did your usage of app name at use start time went as expected?	Yes/No/I am not sure	Single choice	Expectation
How was your last usage session of app name at use start time?	MOS 1 to 5 with a color scale from red to green	Slider	Application’s QoE
What action were you trying to accomplish?	CONSUME content, SHARE or create content, READ text message, WRITE text message, CONTROL an app (start/stop music), VIDEO call or AUDIO call	Multiple choices	Task
Did your last usage of app name at use start time meet your expectations?	MOS 1 to 5 with a color scale from red to green	Slider	Expectation MOS
If something went wrong, please tell us more about it	Text	Free text entry	Anecdotal

### User’s expectation and QoE

Even though the expectation is pointed out in QoE models, its assessment in Internet-based services is rare. In 2012, Sackl et al. [20] proposed an experiment to test user expectation and QoE on wireless 3G connection versus an ADSL Internet access. The participants were directed to test various internet usage scenarios, mainly browsing websites and playing videos on a laptop. The authors modulated the QoS and provided on-screen the Internet connection type label (i.e., wireless 3G or wireline ADSL) to the user. They showed that expectations, as QoE, are relying on usage scenarios and applications. However, their lab-based study did not take into account how many of their participants used a 3G or ADSL in their day-to-day life. In their later work in 2014, Sackl et. al. [2] were able to improve two Web QoE models (Google Maps and file download) using expectation related data gathered via questionnaires before their in-lab experiment. After each test, the participants rated the experienced quality using a 5-point Absolute Category Rating (ACR) scale. The two models integrated two expectation types: desired expectation, for the Google Maps application and adequate expectation, for downloading online files. The desired expectation is mostly constant over time, contrary to the adequate expectation prone to change depending on the current context [21]. The other input in the model was the downlink bandwidth (DLBW). The authors targeted the MOS rating given by their participants; as they integrated users expectations in QoE assessment, they increased the MOS prediction accuracy in their models. Sackl et al. [22] in 2017 investigated user expectations and QoE in the context of networked multimedia. They showed how QoE could be integrated into QoE research. They focus on expectations before a task. Their experiments were lab-based in a controlled environment. The expectation is often influenced by the novelty of the user’s context and its past experiences (i.e., fulfilment or disappointment).

The majority of the past work was in-lab, without taking into account the importance of external contextual factors influencing the QoE. The “in the wild” studies were not conducted unobtrusively. They focused on network QoS (i.e., flow size and throughput) and did not integrate user behavior in the application (e.g., time spent and task to accomplish) and expectation. Given the state of the art, we are in a unique position to provide insight to enable accurate modeling and prediction of mobile QoE in a living lab setting in the users’ daily life contexts.

## The approach: user study

To find the factors affecting smartphone applications’ QoE “in the wild” and to create models to predict QoE, we conducted a user study. We present our study protocol (“Study protocol” section), then we describe the tools enabling us to collect the data (“Ecological momentary assessment (EMA)/MOS” section and “Smartphone-based data Collected” section). Finally, we summarize the data acquired in the study (“Collected data summary” section).

### Study protocol

In our study, participants rated their smartphone application usage QoE in a minimally intrusive manner on their Android OS smartphones using our application. Adult participants were recruited via an ad campaign on the University of Geneva (UNIGE), Centre Universitaire d’Informatique mailing list. We selected the ones using Android OS smartphones for the longest and using a set of apps from different categories that are highly interactive and popular. The picked applications on the Google Play Store were respectively in the top 5 for their categories: messaging, social network, music, navigation, and Internet browsing. The categories correspond to the listing on the Google Play Store. They are Google Chrome, Google Maps, Spotify, Instagram, Facebook, Facebook Messenger, and WhatsApp. Those applications are used on millions of devices. Our selection was based on minimizing the effect created by the introduction of new applications to our participants (i.e., limiting bias) and to maximize our recruiting pool. In our set of selected applications, we found common tasks available to the user, even in applications from different categories. For example, it is possible to share content in all the applications. However, only some allow listening to audio content: Spotify (song), WhatsApp (voice message), Messenger (voice message), and Facebook (song in the timeline). We recruited 38 participants (15 females, two non-disclosed) along November to December 2018 (P1-P38). The study ran for 28 consecutive days in two languages common at UNIGE, to allow more people to join. The participants were invited to install our homemade application mQoL-Lab which integrated our data logger mQoL-Log.

### Ecological momentary assessment (EMA)/MOS

EMAs originated in psychology as a momentary assessment of an individual’s state or emotion [23], hence limiting errors caused by memory effect on the ratings. EMAs were used to gather QoE ratings “in the wild” [10]. We implemented EMAs in our mQoL-Lab via surveys after a specific application usage detected by mQoL-Log.

**Fig. 1:**
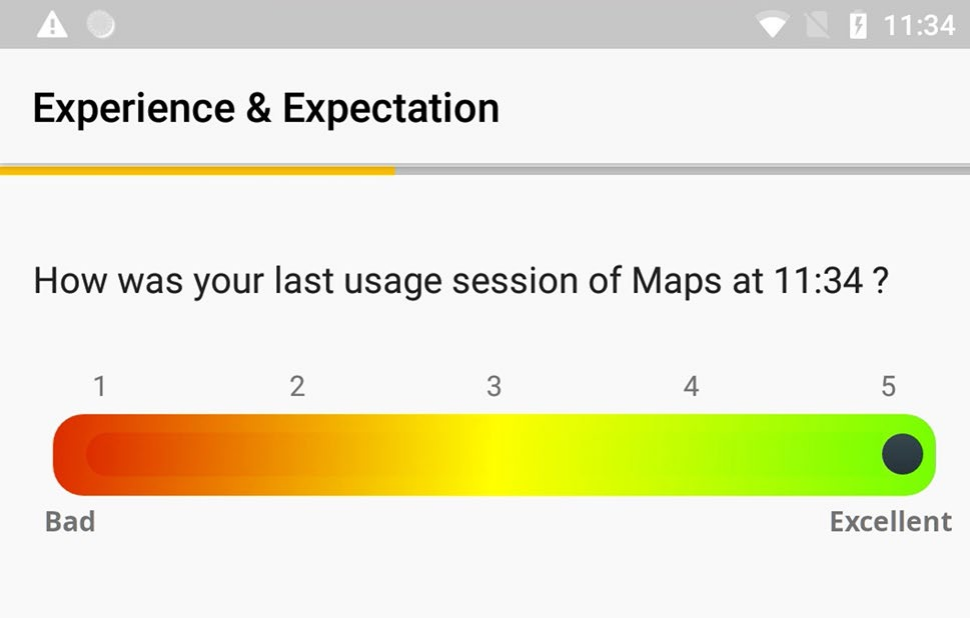
User selecting QoE=5 on the scale after using (Google) Maps

The number of EMAs triggered per day was limited to 12. EMAs were only launched in the waking hours (i.e., outside 21:00 to 7:00), and the time interval between two consecutive EMAs was set up to 20 minutes. If the previous EMA was not filled when a new one is triggered, the previous one was dismissed. Users replied to the surveys by clicking on the notification at the screen’s top. The EMA questions and their possible responses in our study are available in Table 1. As shown below, some questions requested binary responses, other multiple choices. Finally, for the QoE rating, we used the Mean Opinion Score (MOS) [24], a subjective rating scale from 1 to 5 mapped to the following rating: poor (1), bad, fair, good, excellent (5). The slider on the screen allowed for continuous rating as it offered a higher definition on the nearest target (e.g., 3.5 is between ’fair’ and ’good’, 3.8 is close to ’good’ for acceptability of a service [6]). On the application level, the slider scale contains a two decimals digits precision (e.g. 1.00 to 5.00). We round this information to one decimal, as the participant does not see this level of detail on his/her screen. Figure 1 represents a user selecting 5 on the MOS scale. The expectation questions are new; in past work on expectation and QoE [22, 25], study participants were asked to rank affirmations about their expectation of specific Web service (e.g. “What do you expect from a Video on Demand Provider?”). The provided questionnaires were modified for our use case, as they advised in their work. We diverged by assessing the user’s expectation fulfilment after the experience. Hence, we investigated whether or not their expectation impacts their experience. If so, does this factor impact QoE modeling. In past studies, all the questions of the EMA were the same regardless of the type of application, while our third EMA question allowed us to understand the user’s purpose in the app. We decide to reduce the number of classes by labeling each sample in terms of “High” QoE MOS above 3.5 included and “Low” QoE MOS below 3.5, as recommended by the ITU-T [25] and previously employed by Schatz et al. [6].

### Smartphone-based data collected

The mQoL-Log background phone logger [26] collected various timestamped data. Our first trial study for smartphone applications’ QoE [27] indicated the importance of the user’s actions to be accomplished inside an application. The Table 2 includes the data collectors’ descriptions and triggers. QoS is a part of QoE [1]. Hence, the network related data were important. Once the participants finished their application usage session, mQoL-Log performed a network reachability test, also known as *ping*, to the application server corresponding to the app. A ping provides Round-Trip Time (RTT) [ms] data as an indication of the QoS level. The RTT is the time that takes a packet to go from the client through the network to the host, including the time for the host reply to arrived at the client. The ping is done six times, and the first is discarded in case it was subjected to a DNS resolution. We set up a ping time out threshold of 60 seconds. mQoL-Log stopped the test if the threshold was met. The pings were executed at the beginning of the application usage, for which the QoE/EMA was being triggered. Besides the RTT, another important QoS feature is the overall network traffic of the smartphone. To gather the network connection flows information (TCP and UDP, source IP, destination IP, ports, TCP states), we collected the output of the Linux *netstat* command, which did not need root access (see details in “Features” section). We purposely recorded the ones proven by the literature (in-lab and “in the wild” on other platforms) to be an accurate indicator of smartphone application’s QoE.

**Table 2:** mQoL-Log: Background Logger Data Collection

Name	Definition	Trigger
Network	WiFi level, WiFi BSSID, WiFi SSID, WiFi interface speed, Cell ID, Cell operator, Cell strength, Cell radio access technology, Cell network code, Internet connection status, netstat (TCP network statistics), IP address, Cell bandwidth up and down stream, proxy information, domain name. Number of packet and bytes sent and received on wireless interfaces, DNS’s IP address, routing table information	Changes in network connection state and during user app usage
Ping/RTT	Active probing of the application used Internet server. A ping is executed 6 times. We derive statistics (mean, stdev, and variance) from this test.	When the app usage session starts
Battery	Battery state (e.g., charging, full, discharging), battery level, battery temperature	Changes in battery state
App name	Application name on the user screen	Changes of the application on the screen
Physical activity	User physical activity from the Google play services activity (still, tilting: between two states, in-vehicle, on a bicycle, on foot, running)	Changes in the user activity
Touches	Number of user touches on the screen and duration during a usage session	Screen event-based: a new smartphone session

### Collected data summary

**Table 3:** Study participation raw metrics

User ID	MOS [mean ± sem]	Trig [n]	Ans [n]	$P_{rate}$ [%]
1	5.0 ± 0.02	131	27	20.6
2	3.88 ± 0.52	133	121	90.9
3	3.84 ± 0.62	313	271	86.5
4	3.88 ± 0.77	64	52	81.2
5	4.91 ± 0.46	309	290	93.8
6	4.96 ± 0.21	178	172	96.6
7	4.73 ± 0.75	92	87	94.5
8	4.0 ± 0.0	193	136	70.4
9	4.98 ± 0.16	213	196	92.0
10	4.91 ± 0.47	336	324	96.4
11	3.81 ± 0.55	230	200	86.9
12	4.98 ± 0.19	159	123	77.3
13	4.51 ± 0.87	347	318	91.6
14	4.86 ± 0.39	241	215	89.2
15	4.19 ± 0.67	380	120	31.5
16	4.9 ± 0.44	136	22	16.1
17	4.68 ± 0.53	251	145	57.7
18	4.91 ± 0.52	230	207	90.0
19	4.57 ± 0.77	298	223	74.8
20	4.24 ± 0.65	354	131	37.0
21	4.79 ± 0.57	150	139	92.6
22	4.96 ± 0.25	334	266	79.6
23	4.97 ± 0.33	289	185	64.0
24	4.95 ± 0.25	269	142	52.7
25	4.99 ± 0.21	504	369	73.2
26	3.59 ± 0.62	228	221	96.9
27	4.83 ± 0.59	126	110	87.3
28	4.9 ± 0.45	299	264	88.2
29	4.97 ± 0.2	318	277	87.1
30	4.84 ± 0.5	289	164	56.7
31	4.95 ± 0.33	232	188	81.0
32	4.93 ± 0.31	82	71	86.5
33	4.96 ± 0.4	184	160	86.9
34	4.0 ± 0.0	40	37	92.5
35	4.62 ± 0.68	78	72	92.3
36	4.79 ± 0.58	167	88	52.6
37	4.57 ± 0.76	184	134	72.8
38	4.05 ± 0.23	62	21	33.8
ALL	4.61 ± 0.43	222 ± 104	166 ± 89	75 ± 23
$\sum$		8445	6308	74.7

The age distribution of the 38 participants is as follows. Twenty-two were young adults (three between 18 and 20 year-olds and 19 between 21 and 29 year-olds), followed by ten participants between 30 and 39 year-olds, two between 40 and 49 year-olds, two participants between 50 and 59 year-olds and two non-disclosed their age.

As the number of EMA per day was limited to 12, only a maximum of 336 ratings could have been collected per user. A minority of participants (N = 5) did use the designed applications enough to trigger all the possible EMA per day. The participants did not respond (’Ans’) to all the triggered (’Trig’) EMAs. We obtained an average rate of assessments $P_{rate}$=75 ± 23% (where $P_{rate}$ is the number of provided ratings /number of triggered EMAs per participant).Table 3 specify the mean ± std MOS score of the participants and their $P_{rate}$. Only 24 participants obtained a $P_{rate}$ higher than the aggregated $P_{rate}$. We collected 6308 EMAs. We obtain 6086 exploitable EMAs after filtering the incomplete and erroneous EMAs (e.g., incomplete answers in one of the first two questions), we remove 3.5% of EMAs.

**Fig. 2:**
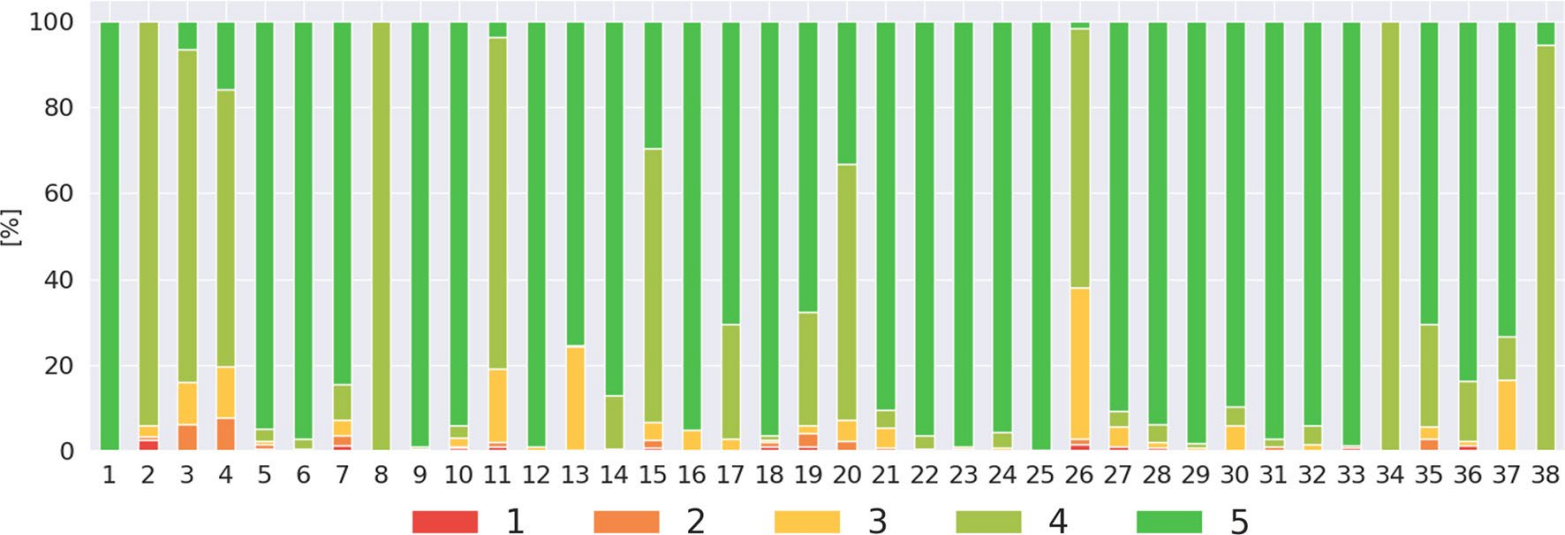
Application usage QoE/MOS rating distribution per participant

We round up the distribution of the application usage rating to the closest integer value and present the result in Fig. 2. The aggregated distribution of those QoE rating is as follows: 0.39% of 1, 0.89% of 2, 5.03% of 3, 20.22% of 4, and 73.45% of 5. The ratings display a high imbalance in the dataset. The prevalence of “High” QoE MOS is 93.5% and 6.5% of “Low” QoE for D38.

**Fig. 3:**
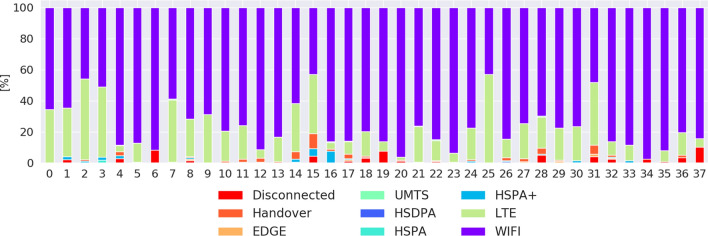
Network connectivity distribution per a participant

The network connection type distribution of our dataset is available in Fig. 3. We defined handover as a change in the networking technology (e.g., cellular to WiFi, WiFi to cellular and EDGE to LTE). The “Handover” label covers the Internet connection transition the cellular network technologies (e.g., LTE to HSPA+; horizontal) and between WiFi and cellular network (vertical). Our application collected samples whenever the phone was used, including samples over cellular networks during the participants’ commute and other mobility events. More than half of our dataset (61%) was composed of QoE rating on a WiFi connection, followed by 31% on LTE, 2% handover, 3% disconnected, 2% on HSPA+ and the last 2% on EDGE, UMTS, HSPA, and HSDPA.

**Fig. 4:**
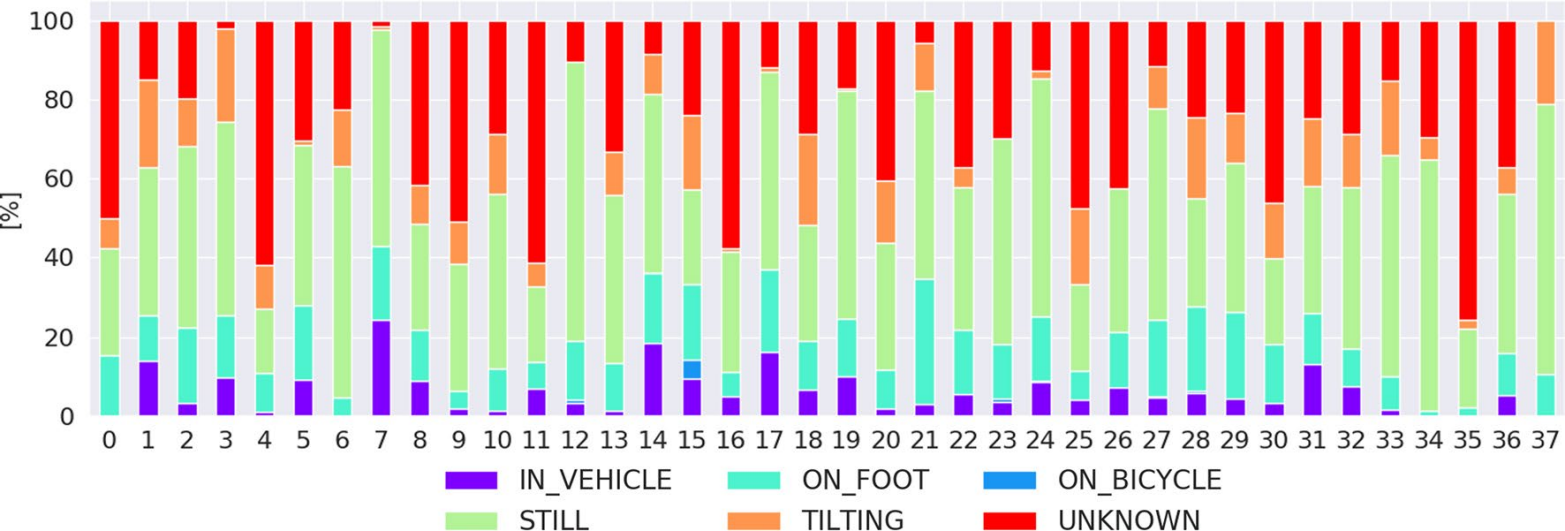
Physical Activity distribution per participant

We found that the participants’ physical activities during the application to be “still” at 52%, followed by “on foot” with 20%, “tilting” at 16%, “in vehicle” with 11%. The per user distribution of activity is in Fig. 4.

The distribution of the intended action to accomplish was as follows: 42% of “consuming content”, 24% “reading and writing messages”, 18% “reading messages” only, 6% of non-labeled and 5% “write” only”. The remainder of the dataset contains the actions like “audio”, “video”, “control application”; in total less than 2%. “Low” QoE (MOS $\in$ [1 to 3.5]) was more present for the non-labeled actions like “consuming content” than for “reading and writing messaging” and own single actions “writing messages” and “reading messages”. “High” QoE was more prevalent for more actions for all applications.

We observed the same rating behavior from the different age categories, except for the oldest (50 to 59 years old) - they all rated “High” QoE higher than 90% of the time. The latter rate “High” QoE only in 84% of their application usage. “time to reply” is defined as the amount of time between the end of application usage and the moment when the participant started to reply to our EMA, overall the mean is 14.85 ± 0.95 minutes. The oldest group provided the rating the fastest, 94% lower than the mean “time to reply” overall categories. The 30–39 group answered 91% under the same threshold. The younger groups followed, 89% for the 21–29 and 84% for the 18–20. The 40–49 group took longer to answer. Only 77% of their ratings were given under the threshold. On average, a study participant took more time to rate “Low” QoE (1.02 ± 0.26 min, mean ± sem) than “High” QoE (0.36 ± 0.05 min).

We defined the “screen session” as the amount of time between the screen turning on, and the screen turns off automatically (system timeout) or from a user’s action (manual locking). Several applications are generally used one after another during those sessions. We computed the mean time spend inside the same screen session as 6.95 ± 0.2 minutes. On average, the users spent 2.34 ± 1.53 minutes in the selected applications of this study. On average, a participant rated 166 ± 89 application usage over the study period, overall 6 ± 3 per day. We name D38 the fully cleaned collected dataset.

The youngest group spent more time than any other groups (16.67 ± 2.46 minutes) in a session. Surprisingly, the oldest group (50–59 years old) spent 11.12 ± 1.74 minutes, coming second. They are followed by the 21–29 group with 6.76 ± 0.27 minutes. The 30–39 group spent on average 5.39 ± 0.47 minutes in “screen session”. Finally, the 40–49 group spent less time in the sessions (4.89 ± 0.57 minutes).

We investigated the users’ expectations distribution from their answers to the first EMA question. Overall, only in 2% of application usage session participants were not sure about their expectations. We found that 95% of their application usage session went as expected. In “Low” QoE application’s session, 76% was unexpected. In “High” QoE application’s session, we found 96% of expectation matched. We found a moderate positive relationship [28] with a correlation between the expectation and the QoE rating of 0.595.

**Fig. 5:**
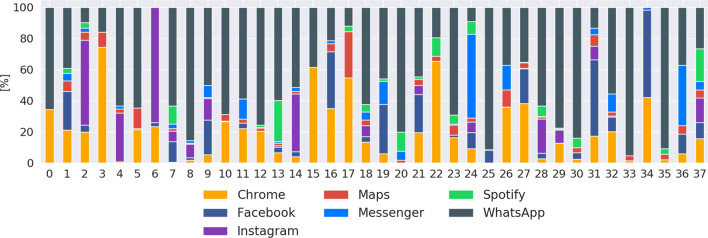
Applications distribution per participant

## Building QoE prediction models

Our goal is to predict the “High” or “Low” QoE of smartphone application usage based on the on-board data collected from the smartphones labeled by the participants’ QoE ratings. The latter is used as a ground truth. We formulate it as a classification problem. We start by selecting features from our collected dataset (Table 2). The features are the ones influencing the user while in an application usage session, and hence they are the input data of our model. We motivate our feature selection in the following “Features” section. We apply the most appropriate learning practices during our modeling. We split our data into training, validation (used for classifier hyperparameters’ optimization), and testing dataset before any oversampling (no-leakage). We explore the features’ importance in our prediction models. The importance is provided by the eXtreme Gradient Boosting library [29] powering the models. For each model, we report its predictive accuracy, AUC, and recall to evaluate its performance in “Results” section. For the previously listed metrics, values closest to 1 are optimal.

**Fig. 6:**
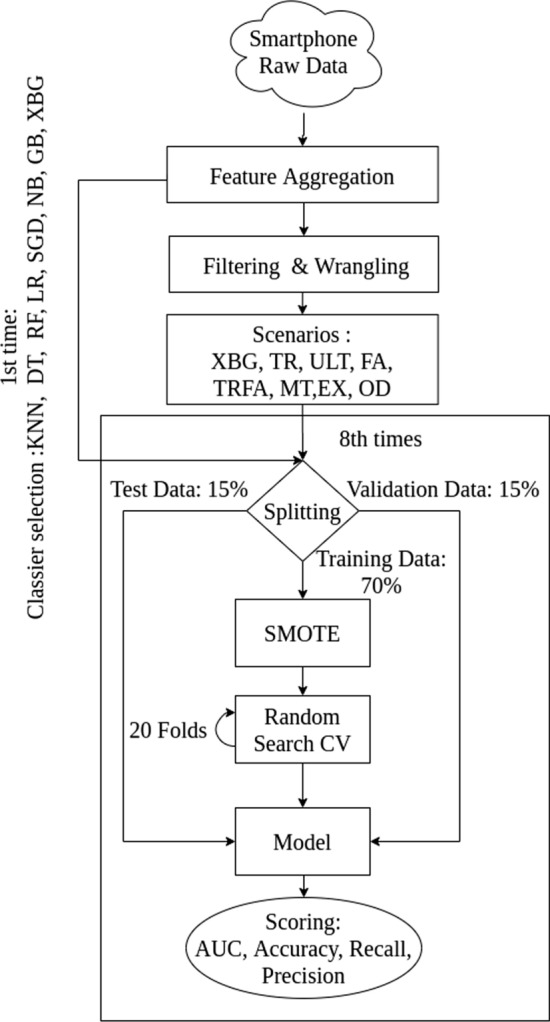
QoE Modeling Process Pipeline

Figure 6 summarizes our process pipeline. We went through the pipeline eight times. The first time was to select the best machine learning classifier for our QoE prediction model. The other times, it was for building models with different input features and data aggregation methods.

### Features

This section described part of Fig. 6, the data aggregation, filtering, and wrangling blocks. From the different data collected in the background, we select and aggregate features centered on the beginning time of the application usage session of interest, hypothesizing that these features relate to user QoE. The time-based aggregation was done using a time window of two minutes centered on the QoE rating. This time window is selected based on the average time spent by users in the applications selected in the study from our dataset (2.34 ± 1.53 [min]). If data was unavailable for a feature, we add one more minute ( ± 30 seconds before and after the current time window) until data were found. During the reprocessing of our dataset, we filter the data collected via mQoL-Log to remove incomplete and erroneous data (e.g., application usage session of ten hours).

We pick the networking features in our dataset. Therefore, we perform feature engineering to extract information from multiple features, e.g., knowing the IP address allowed us to know if the application operated over IPv6 or IPv4. We extract the TCP states’ distinct count for each app usage. The list of all the network features used in the model is presented in Table 4. mQoL-Log recorded the network changes as they occurred, even during the application usage. Throughout the aggregation process of the network features, the potential handover information was encoded. Further, we aggregate the QoE rating with the battery state, the application session duration, the application name, and the task to fulfill the need of the participant in the application. Additional external factors influencing QoE are context-based. Hence, we select the features with high context (e.g., physical activity).

**Table 4:** Network features as collected via mQoL-Log during application usage

Features	Description	Type/Unit
is_connected	Connection status from Android OS	Boolean
Connection type	Network connection type (Fig. 3)	Categorical
WiFi level	Signal strength of connected Access Point	Float/dbm
WiFi speed	WiFi interface speed	Float/Mbps
Cell strength	Signal strength of connected cell tower	Float/dbm
Cellular down bandwidth	Cell downstream bandwidth	Float/Kbps
Cellular up bandwidth	Cell upstream bandwidth	Float/Kbps
win_div_net	Aggregation window for network events around the application usage time	Int/minutes
rxt_packets_time	Packets received per seconds during win_div_net	Float/pps
txt_packets_time	Packets sent per seconds during win_div_net	Float/pps
rxt_bytes_time	Bytes received per seconds during win_div_net	Float/Bps
txt_bytes_time	Bytes sent per seconds during win_div_net	Float/Bps
$RTT_{mean}$	Mean Round-Trip Time of the 5 pings	Float/minutes
$RTT_{variance}$	variance Round-Trip Time of the 5 pings	Float/minutes
Netstats: TCP states count during win_div_net	LISTEN, SYN-SENT, SYN-RECEIVED, ESTABLISHED, FIN-WAIT-1, FIN-WAIT-2, CLOSE-WAIT, CLOSING, TIME-WAIT, CLOSED [30]	Int/Categorical

**Table 5:** QoE prediction: metrics on the validation datasets for multiple common classifiers

Classifier	AUC	Accuracy [%]	Precision [%]	Recall [%]
K-nearest neighbours (KNN)	0.796 ± 0.036	0.758 ± 0.014	0.969 ± 0.007	0.765 ± 0.014
Decision tree (DT)	0.612 ± 0.056	0.913 ± 0.016	0.952 ± 0.01	0.955 ± 0.012
Random forest (RF)	0.758 ± 0.029	0.908 ± 0.014	0.955 ± 0.01	0.946 ± 0.01
Logistic regression (LR)	0.775 ± 0.024	0.823 ± 0.008	0.967 ± 0.005	0.839 ± 0.01
Stochastic gradient descent (SGB)	0.773 ± 0.027	0.84 ± 0.006	0.964 ± 0.006	0.86 ± 0.005
Naive bayes (NB)	0.685 ± 0.019	0.087 ± 0.012	0.956 ± 0.039	0.021 ± 0.005
Gradient boosting (GB)	0.774 ± 0.027	0.916 ± 0.013	0.951 ± 0.011	0.959 ± 0.007
eXtreme boosting gradient (XBG)	0.816 ± 0.017	0.931 ± 0.01	0.952 ± 0.009	0.975 ± 0.006

### QoE/MOS classification

Following the work of [31], we normalize the QoE rating values per each user. We use one-hot encoding on our categorical features (i.e., network type, application name, physical activity, and task) to prepare them for the classifiers. We follow a classic machine learning method by randomly selecting stratified 70% of our data as our training dataset. The resulting 30% is split into two to obtain our validation dataset (15%) and testing dataset (15%). We conduct a randomized search cross-validation ($cv=20$) to optimize our model parameters. That means that the 70-15-15 split has been run 20 times by repeating the random selection of our training, validation and testing dataset, hence covering our entire dataset. This is called “random permutations cross-validation (shuffle and split)” [32]. The distribution of “High” and “Low” QoE ratings are preserved in the validation and testing dataset.

After each split, as presented in Fig. 6 we apply SMOTE [33] on the training dataset to overcome the imbalance issue via over-sampling. We perform down-sampling in our pre-analysis. The models trained with smaller datasets are not able to generalize as the ones after SMOTE. The training dataset has 50% of “High” QoE labels (n = 3981, no rating was lost), and 50% of “Low” QoE labels (n = 3981); we gain 3702 “artificial” ratings. We scale our training dataset to remove the mean and scaled to unit variance, as some classifiers (e.g., K-nearest neighbors) have issues with data of different unit sizes. This scaler is used on the validation and testing dataset.

### Classifier selection

The process in Fig. 6 is done in this section.

In the first pass through our process in Fig. 6, we investigated the most accurate classification algorithm for our goal. We ran a *candid* (non-optimized) 10x fold cross-validation on our training and validation dataset to select the algorithm with the best performance for our classification problem between: k-nearest neighbors (KNN), decision tree (DT), random forest (RF), logistic regression (LR), stochastic gradient descent (SGD), Naive Bayes (NB), gradient boosting (GB) and eXtreme Boosting Gradient (XBG).

We selected the Area Under the Curve (AUC) [0-1, no dimension] as the metrics to find out the more accurate classifier of our list. It expresses how accurate a model can distinguish between classes (e.g., classifying the classes correctly with minimum confusion). It measures the entire two-dimensional area underneath the receiver operating characteristic curve (ROC). The ROC is a graph (*x*-axis: False Positive Rate,* y*-axis: True Positive Rate) presenting the classification model’s performance. The “accuracy” is the fraction of predictions our models found out right, defined for binary classification as the sum of true positives and true negatives divided by the sum of true positives, true negatives, false positives, and false negatives. The “precision” is defined in % as the true positives divided by the sum of true positives and false positives. It is the proportion of actually correct positive identification. The “recall” is defined in % as the true positives divided by the sum of true positives and false negatives.

Table 5 shows the AUC of our classifiers on the validation dataset. We find that the XGB classifier performs better in the AUC, accuracy and recall metrics. Hence, we select XGB as the base classifier to predict the most accurately “High” or “Low” QoE in this work. XBG has been the most accurate algorithm used in classification problems based on tabular datasets. Boosted tree algorithms, as XBG, have shown their performance on QoE prediction in the past work [34].

We tested eight data filtering and wrangling scenarios (“Candid Model (XBG)” to “On-device prediction (OD)” section ) assuming we can obtain better prediction results on our D38 dataset before applying SMOTE. The same classifier is used in all the scenarios. This step of the process is shown in Fig. 6 in the block “Model”. We examined in section 5 the performance of all the models.

### Candid model (XBG)

We run the same machine learning method as described in “QoE/MOS Classification” section, with the same features used in [3]. After the aggregation, we exploit the 6086 ratings (D38) to train the model before SMOTE. We use this model as our referent model (*XBG*), to compare the models constructed with the filtered D38 dataset in the following scenarios. On the contrary, from the more accurate model presented in “Classifier selection” section, *XBG*’s hyperparameters are optimized. We present its performance in Table 5.

### Filter “time to reply” (TR)

Like in “Candid model (XBG)” section, we use the same features, but in this scenario, we filter our dataset D38. Specifically, we remove the ratings were the user did not answer to the EMA notification after a specific time threshold. We determine the threshold per a dataset, as the mean overall response time after the EMA was triggered. We remove 953 observations with a threshold at 14.85 minutes, leaving us with 5133 ratings with a similar distribution 93.51% of “High” QoE and 6.49% of “Low” QoE. Our hypothesis was as follows. Participants’ ratings are influenced by the time difference between the app use and its rating (EMA). Hence by removing the ratings distant from the events, we anticipate achieving better performance for our model. We present the distribution of the time to reply per user in Table 6. The maximum times are so high as participants would reply in the morning to a notification from the past day.

**Table 6:** Times to Reply (TR) to EMAs per user

User ID	Time to reply mean ± sem [min]	Minimum [min]	Maximum [min]
1	32.11 ± 20.86	0.02	641.41
2	10.84 ± 10.86	0.02	666.18
3	7.14 ± 5.73	0.02	524.20
4	0.58 ± 0.14	0.01	4.11
5	12.25 ± 12.4	0.01	870.12
6	18.41 ± 16.89	0.01	774.19
7	3.5 ± 1.93	0.03	140.19
9	1.39 ± 0.87	0.02	50.16
10	6.64 ± 7.15	0.02	544.88
11	6.85 ± 2.64	0.01	112.61
12	14.53 ± 7.8	0.01	511.51
13	40.05 ± 30.29	0.03	1282.35
14	3.66 ± 2.7	0.01	203.57
15	21.85 ± 12.6	0.02	774.97
16	32.86 ± 31.29	0.01	1569.44
17	12.6 ± 11.96	0.01	755.89
18	27.47 ± 11.27	0.01	234.51
19	31.99 ± 21.11	0.03	813.43
20	6.76 ± 7.23	0.01	592.61
21	1.17 ± 0.86	0.01	59.88
22	2.45 ± 1.46	0.02	80.83
23	11.2 ± 8.48	0.02	515.09
24	5.96 ± 2.4	0.01	62.58
25	11.39 ± 11.19	0.02	543.64
26	23.41 ± 21.07	0.01	1006.80
27	28.51 ± 22.69	0.03	1088.75
28	6.32 ± 2.39	0.05	111.54
29	5.83 ± 5.53	0.02	553.98
30	6.56 ± 7.06	0.02	583.67
31	12.44 ± 8.96	0.01	551.27
32	15.33 ± 6.42	0.03	140.73
33	15.47 ± 9.25	0.01	551.64
34	15.84 ± 13.34	0.02	802.23
35	15.72 ± 14.33	0.01	794.24
36	4.98 ± 2.26	0.01	105.39
37	65.23 ± 42.98	0.03	1732.94
38	13.13 ± 12.54	0.01	768.73
D38	14.85 ± 0.95	0.02 ± 0.01	577.89 ± 0.54

### Unlabeled tasks (ULT)

As previously shared, 6% of the samples of D38 have unlabelled tasks (i.e., the unanswered question for “What action were you trying to accomplish?”) for a given application. We try to fill those samples with the most common task a user-selected per application during the study for D38. We hypothesize that the influences of those samples could allow for a more accurate predicting model. The most common task per application are as follow: WhatsApp and Messenger are used to “READ” and “WRITE” messages. Spotify for consuming music. Chrome, Instagram, Maps, and Facebook were used to “CONSUME” different types of content. We attempt to derive a QoE model assuming that, as presented later in the results section.

### Filter features aggregation time (FA)

We propose to execute the same method used in Filter ”time to reply” section. However, instead of filtering the time delta between the application usage and its rating, we filter the dataset (D38) based on the time between the EMA and the times the aggregated features were generated (e.g., win_div_net feature for the network data collection service). As Android OS was terminating our data logger from time to time, and since not all users allowed the application to upload all the collected data to completion, we remove the samples in which the features were collected too far-off the rated event. We define the far-off threshold with a lower bound and higher bound as $\Delta$ $t_{mean\pm std}$ for each data collection service, $\Delta$t was the time difference between the event and the time of collection for the data collection services as described in Table 2. Each threshold was applied to its corresponding data collection services from Table 2. It reduce the dataset to 4082 samples (2003 samples were removed). Table 7 shows the respective thresholds in minutes per data collection service. It represents the mean ± standard error (sem). We do not test other cut-off thresholds.

**Table 7:** Aggregation Features (FA) Threshold in minutes for each mQoL-Log service

mQoL-Log lollection services (Table 2)	*FA*38 [min]
Network	0.56 ± 0.50
Ping/RTT	3.87 ± 0.53
Battery	0.11 ± 0.03
Physical activity	4.03 ± 0.22
Touches	0.56 ± 0.50

### Merged filter replies (TR) and features aggregation (FA) time (TRFA)

We removed samples that matched two filters from two different blocks (aggregation and filtering) of our process pipeline (Fig. 6): the feature time aggregation (FA) from “Study limitations” section and the time to the replied threshold (TR) as described in “Discussion” section. For both filters, the same previous thresholds are respectably applied. It reduced our dataset from 6086 samples to 3701 samples. The same features as before are used to train the model.

### Meta-features selection (MT)

**Table 8:** Aggregated Features Importance from *TR*, *FA* and *ULT*

Feature name table 2 , 4	Importance mean ± sem [%]	On device android feasibility
Task	38.37 ± 2.64	$\checkmark$
Physical activity	9.03 ± 0.53	$\checkmark$
Application name	12.04 ± 0.77	$\checkmark$
Battery level	2.94 ± 0.12	$\checkmark$
Cell strength	4.70 ± 0.12	$\checkmark$
Network type	3.05 ± 0.45	$\checkmark$
is_connected	2.15 ± 0.10	$\checkmark$
Cellular down bandwidth	1.10 ± 0.11	$\checkmark$
Cellular Up bandwidth	1.75 ± 0.12	$\checkmark$
Aggregated packets traffic stats	1.20 ± 0.10	
win_div_net	3.44 ± 0.12	$\checkmark$

From our previous modeling attempts (“Candid model (XBG)” section– “Merged filter replies (TR) and features aggregation (FA) Time (TRFA)” section), we evaluate the most predictive features and only used this subset for training our model. We aggregate all the models generated by the 20 folds with random grid search for each of our previous attempts: filtering based on the time difference between the event and the participants’ annotation (*TR*), filtering the features based on time of collections (*FA*) and mapping the non-labeled task (“Unlabeled tasks (ULT)” section, *ULT*). We generate a total of 60 models ($3*20$ folds). We extract the importance of each feature for each model and computed the mean ± std error for each. We select only the features with average importance higher than 1% (arbitrary threshold). The importance is available through the XGBoost library used to train the model. Table 8 contains the features and their importance. “Aggregated packets traffic stats” represent the aggregation of the different features from the packet statistics (i.e., the features with the suffix “_times” in Table 4).

### Expectation (EX)

As we found a moderate correlation between the expectation and the perceived QoE (“Collected data summary” section), we propose to use this information as an additional feature in our new *EX* model. We use the same base features as presented in *XBG* (“Candid model (XBG)” section). The expectation is based on past application sessions, e.g., prior knowledge about context and an event (i.e., “Low” QoE on WhatsApp when connected to the university WiFi) [22].

### On-device prediction (OD)

We selected the features that could be used to predict QoE directly on the device, transparently for the user. Some previously used features generated via feature engineering during aggregation (i.e., packets traffic stats) or duration of application usage are not information the Android OS application can obtain. Hence, we decided to base our features for this model with the ones from Table 8 and the features used to train our candid model.

At the time of this writing, Android OS version 10 has been released in November 2019. It includes new security measures. It is not possible to access the “netstat” command output as before, and the priority of background service execution has been modified. A new limitation was introduced with Android 10, the long-running background network services are restricted by the system. Hence, we can not use an active ping probe. We remove the features that could not be integrated into an on-board smartphone model. The on-board accessible features were as follows: battery level, user physical activity, application, the task in the application, Android network manager “is_connected” attribute, network connection type, WiFi level, WiFi speed, cell strength, cell bandwidth up and downstream.

Table 9 summarized all the features used in the previous scenarios and with the closest related work, i.e., building a smartphone application’s QoE prediction model [17]. As the *XBG*, *TR*, *FA*, and *ULT* models share the same features but used several aggregations and filtering methods, we group them into one group *G* for the figure clarity.

**Table 9:** Model’s features per scenario

Perspectives	Features	${G}^{1}$	*MT*	*TRFA*	*EX*	*OD*	Casas et. al. [17]
Context	Physical activity	$\checkmark$	$\checkmark$	$\checkmark$	$\checkmark$	$\checkmark$	
	Location						$\checkmark$
User	Task	$\checkmark$	$\checkmark$	$\checkmark$	$\checkmark$	$\checkmark$	
	Expectation				$\checkmark$		
	Duration user session	$\checkmark$	$\checkmark$	$\checkmark$			
System	Application name	$\checkmark$	$\checkmark$	$\checkmark$	$\checkmark$	$\checkmark$	$\checkmark$
	Battery level	$\checkmark$	$\checkmark$	$\checkmark$	$\checkmark$	$\checkmark$	
	Cell strength	$\checkmark$	$\checkmark$	$\checkmark$	$\checkmark$	$\checkmark$	$\checkmark$
	Network type	$\checkmark$	$\checkmark$	$\checkmark$	$\checkmark$	$\checkmark$	$\checkmark$
	IP version	$\checkmark$	$\checkmark$	$\checkmark$	$\checkmark$		
	is_connected	$\checkmark$	$\checkmark$	$\checkmark$	$\checkmark$	$\checkmark$	
	Cellular down bandwidth	$\checkmark$	$\checkmark$	$\checkmark$	$\checkmark$	$\checkmark$	
	Cellular up bandwidth	$\checkmark$	$\checkmark$	$\checkmark$	$\checkmark$	$\checkmark$	
	Handover	$\checkmark$		$\checkmark$	$\checkmark$		
	Netstats	$\checkmark$		$\checkmark$	$\checkmark$		
	Aggregated packets traffic stats	$\checkmark$	$\checkmark$		$\checkmark$		$\checkmark$
	$RTT_{mean}$	$\checkmark$		$\checkmark$	$\checkmark$		
	$RTT_{var}$	$\checkmark$		$\checkmark$	$\checkmark$		
	WiFi level	$\checkmark$		$\checkmark$	$\checkmark$	$\checkmark$	
	WiFi speed	$\checkmark$		$\checkmark$	$\checkmark$	$\checkmark$	
	win_div_net	$\checkmark$	$\checkmark$	$\checkmark$	$\checkmark$	$\checkmark$	
	Cell ID						$\checkmark$
	Cell Operator						$\checkmark$
	TCP flow ratio						$\checkmark$
	Duration flow						$\checkmark$

## Results

In this section, we summarise our past results from [3] and present the output from our new scenarios (“Candid model (XBG)” section– “On-device prediction” section).

### Overview of previous work

In our past work [3], we had 33 participants and collected 5663 ratings. We build on QoE prediction model named $XBG_{33}$. Its performance metrics are as follows AUC of $0.8388\pm 0.279$ and accuracy $0.939\pm 0.007$%. We derived the importance of the features in the most accurate XBG classifier and found that the duration of application usage, battery level, and QoS features, user’s tasks to be accomplished are relevant (e.g., send text versus consuming content), as well as the user physical activity (e.g., walking) to predict QoE. The participant task, in the application itself, is more important than the application used.

**Table 10:** QoE Models performance on test datasets for each scenario

Scenario	AUC	Accuracy	Precision [%]	Recall [%]
${XBG}_{33}$[3]	0.829 ± 0.028	0.939 ± 0.008	0.953 ± 0.007	0.984 ± 0.004
*XBG*	0.812 ± 0.033	0.938 ± 0.007	0.952 ± 0.007	0.984 ± 0.004
*TR*	0.83 ± 0.033	0.938 ± 0.007	0.953 ± 0.006	0.983 ± 0.004
*ULT*	0.723 ± 0.034	0.929 ± 0.009	0.94 ± 0.009	0.987 ± 0.003
*FA*	0.813 ± 0.038	0.924 ± 0.008	0.94 ± 0.006	0.98 ± 0.008
*TRFA*	0.822 ± 0.041	0.926 ± 0.011	0.944 ± 0.01	0.978 ± 0.007
*MT*	0.801 ± 0.03	0.931 ± 0.008	0.949 ± 0.008	0.979 ± 0.005
*EX*	0.874 ± 0.027	0.956 ± 0.007	0.967 ± 0.006	0.987 ± 0.004
*OD*	0.76 ± 0.037	0.925 ± 0.01	0.95 ± 0.006	0.971 ± 0.01

### Scenarios’ results

We repeat the same process from “QoE/MOS classification” section for all our scenarios, only using the XBG algorithm. Hence, we train eight models with random hyperparameters search with 20 cross-validations, done 20 times to cover the full dataset.

We test the performance of our models with the test datasets. Our results are shown in Table 10. It contains the two main metrics we selected (AUC and accuracy), as well as precision and recall. We want to validate our results statistically. We apply a pairwise t-test to the metrics, as they are normally distributed for all the scenarios. The null hypothesis *H0* is as follows: there is no statistically significant difference between the scenarios’ metrics. Considering that we are making multiple comparisons, we have to use the Bonferroni adjustment to select the correct cutoff to determine whether *H0* has to be strongly rejected. The base *alpha* is 0.05, and adjusted (for 36 comparisons) is $\alpha =0.001$. If the p-values computed are inferior to $\alpha$, *H0* is rejected. Hence the difference between the scenarios’ metrics is statistically significant.

The candid model *XBG* underperforms on both metrics against the results presented in [3], which are negligible (p-values for both metrics superior to 0.1) with differences of 0.017 for AUC and 0.001 for accuracy. Filtering the time difference between the event and the participant rating time (i.e., scenario TR) allowed for more accurate models. The model *TR* scores higher on AUC and accuracy.

The filtering based on the feature time (FA) aggregation window data allowed for similar performance, *FA*’s AUC is 0.001 higher than *XBG*’s. The model created with both filters on the aggregated time and participants’ replies time (scenario TRFA) *TRFA* scored between *TR* and *FA* on AUC and the same pattern repeated for accuracy.

The models created with the unlabeled tasks (ULT) filled by the most common task per app per user (*ULT*) performs the worst on the AUC metric. The meta-features selection filtering (MT) performance is only of 0.801 for AUC and 0.931 accuracy.^1^

Table 8 shows the selected features and their importance. The “user accomplished task” is the most important feature. The on-device prediction model *OD* scored on 0.76 on AUC and 0.925 on accuracy. The “feasible” features selected for this model did not allow for higher performance.

Integration expectation (EX) in our model is beneficial to predict QoE, *EX* scores higher on all the metrics, with an AUC of 0.874 ± 0.027, 0.956 ± 0.007 accuracy, 0.967 ± 0.006 for precision and a recall of 0.987 ± 0.004. We compute the p-value for each metrics compared with the candid scenario result (*XBG*), for all metrics except recall we found $p < \alpha$ ($p_{AUC}$ = 8.900e-08, $p_{accuracy}$ = 8.049e-10 , $p_{precision}$ = 1.415e-08 and $p_{recall}$ = 0.028). Our last result showed that expectation is linked to QoE for interactive application, during a living lab study “in the wild”.

Overall, the better model to predict QoE is *EX*. We compare AUC, accuracy and precision metrics of *EX* to all the other models and found $p < \alpha$. For recall, the p-values from the comparisons with $XBG_{33}$,*XBG*,*TR*,*ULT* and *FA* are inferior to $\alpha$.

## Discussion

In this section, we discuss our findings from building QoE prediction models with several different features, filtering, and aggregation methods. First, we discuss the rating quality and their influence over our models (“Ratings quality” section), then we argue about our features choices and aggregation method (“Features wranglings” section). Then, we discuss the performances and the implementation of our on-device prediction model (“FOn-device prediction” section). Finally, we share our recommendations for smartphone application developers (“Recommendations for the application developers” section).

### Ratings quality

In light of our results, we saw that the annotators’ rating quality varies from one to another. Users **1** and **8**) always provided the same ratings, even if they were rating different applications, as seen by the distribution in Fig. 5. User **25** provided only one rating of “Low” QoE. The previous assumption that those users’ ratings could be discarded to obtain an increase in QoE model performances had been wrong, as showed by our attempt with the *TR* model. A way to solve this issue in our following studies would be to test the participants with fake-EMAs. We could ask them to rate a false application usage (e.g., with a wrong time or wrong application name) and observe if they communicate about the bogus questions.

Filtering the samples where the participants replied much later after the application usage occurred resulted in better models. The threshold for the rating reliability (i.e., the trust in the participant annotation) was an answer provided within 14.85 minutes. The rating’s reliability is taken into account before selecting the participant’s data for training a model. Convincing the participant to provide a rating just after the application usage is challenging.

### Features wranglings

The model *ULT* scores less than the other models, as we see the task accomplished in the application by the user is the most important feature for QoE prediction, as far as we are concerned. The importance of user tasks to accomplish is ranked first in Table 8. Hence, our method to retrieve the unlabeled task samples to train our model was wrong.

The *MT* model, trained with a reduced set of features, obtaining higher accuracy and AUC than *ULT*. The meta-features selection (“Meta-features Selection (MT)” section) based on the features’ importance from the previous model shows that eXtreme Gradient Boosting could automatically select the essential features for building a model.

We compared our features with the ones from the related work [17]. We saw a higher focus on system-based features (i.e., QoS). Their models were solely trained on one application per model, on cellular networks, with high precision QoS data. We used more features based on the user needs in the application and its context.

### On-device prediction

Machine learning prediction is often directly executed in the cloud. But an Internet connection is not always available depending on the user’s context (e.g., mobile connection in a train tunnel is not available). Hence, we built an on-device prediction model to mitigate this context strain. The limited set of features available for making an on-device prediction model does not perform as well as the other models. The netstats command output was of high importance feature in our past models. The knowledge of the current TCP session states and UDP flows made the models score better. Android has many APIs to query the network state, but none of them is fine-grained. We trained another on-device model with ratings provided in the same time-frame as the TR models’ thresholds. We observed the same behavior as before. Namely, the models with filtered response have higher accuracy. In this case (e.g., scenario TR+OD), the model performs worse than the OD model with 0.782 ± 0.027 in AUC, 0.932 ± 0.008 accuracy and 0.952 ± 0.007 precision but it obtains an increase of recall with 0.977 ± 0.006.

On-device prediction resolve issues linked to data privacy, the input information does not leave the smartphone and the XBG model runs directly on the mobile devices. However, it has shortcomings. The device has to be powerful enough to handle a high number of predictions simultaneously when the phone is processing its normal workload (already running on-screen application and background services). It also consumes extra energy and processor time.

### Recommendations for the application developers

The application developer should optimize their application to seamlessly handle “Low” QoE, depending on what the user wants to accomplish with the application. “Low” QoE ratings are higher when the user is ’writing’ and ’tilting’ between physical activity, hence with this information, the developer could provide a better way of inputting text in their messaging application (e.g., proposing predefined short answer from a half screen size touch area). What is essential for an application developer is that with better QoE, a user is more effective; spends less time on the application accomplishing the intended tasks faster, but potentially also uses more features in an application.

We propose three recommendations for the application developer. They firstly should constantly and accurately monitor the current user context. A change in physical activity, battery consumption, network type, or time spent in their application are a great indicator of QoE. Android OS API allows accessing those data in a simple way via APIs. Secondly, they should integrate a mitigation solution in the case of “Low” QoE. If a model as ours is complex to orchestrate, a cheaper solution for a heavy network application can be a simple ping to their server. If the main action in their application is impacted, they should provide real-time information to the user concerning the issue (e.g., notification to retry with a countdown). As we found out, the model built with expectation as a feature performed better (higher AUC, accuracy, recall, and precision); expectation plays a significant role in QoE. Thirdly, the application developer should use common design and usability patterns provided by the OS maker to optimize expectations and, by doing so, QoE.

### Modelling highlights

Quality and the quantity of data is vital in obtaining a representative model. Our study focused on modeling the Quality of Experience of smartphone users, with their provided ground truth and their smartphone’s data. Overall, the data collection tools have to be tested under multiple contexts to limit the loss of data caused by network instability and participant environment. Once the data are acquired, their quality has to be controlled. It is evident from the models created with reliable data e.g., given the higher availability of the ground truth, one can obtain higher scoring models. Hence, during modeling, the features’ selection, data wrangling, and aggregation steps must be carefully executed to limit model building constrains. The human aspects, such as the user expectation, need to be conscientiously included in the experiment design and the later data analysis. For example, the memory from past application used experiences could create bias when the participant assesses its momentary experience. Thus, following those recommendations permit the creation of QoE models from in-the-wild studies data.

## Study limitations

We consider the following study limitations. First, related to the devices used. This study was only possible on Android OS devices, as data collection is more difficult on iOS. We cannot thus generalize our findings for another operating system platform. Additionally, the collection of the number of frames dropped by an application’s UI would have been a plus to understand the hardware status. New security protection and updated background service execution policy are problematic for data collection without root access. The policy occasionally killed our mQoL-Log collection services to reduce the energy consumption on the smartphone. We estimate we have lost 3.5% of valuable data as a result.

The second limitation was in our choice of applications. We did not include high bandwidth need, which was studied by other [4], particularly video QoE consumption on smartphones. The landscape of smartphone applications is evolving each day with new innovative services, modeling QoE for each new application, and their underlying features’ are not a scalable method. Hence, we tried to generalize QoE prediction based on user action within an application. The limitation is that the user’s momentary emotion and stress level can influence the annotation of their application usage, as well as a participant, can rate a “High” QoE application usage negatively because of the content of the application. The participants were told to avoid this effect, but then it could still influence our models. Smartphone operating system (OS) makers created APIs to obtain the user’s context to allow application developers to write immersive “smart” applications. We use those APIs to gather the participant’s context. Hence, we trust the data validity provided by the OS. Another limitation is the EMA’s questions. They could leave room for interpretation. Hence, they should be updated for our next study to remove this undesirable effect. Furthermore, the dataset collected does not contain a representative population. The uneven age group distribution of our study participants is a limitation of this work’s representativeness. We could not make any conclusions based on demographic information. The presented use case, as well as the tools leveraged to collect the data, the smartphone hardware, and the set of participants are specific to our study. As the reproducibility of our results can be challenged, the presented path towards building smartphone application’s QoE models’ is a first step toward accurate models. Overall, this and similar in-the-wild studies are prone to such limitations, and the number of participants (e.g., implying higher cost per a participant), the study’s duration (i.e., much longer and intrusive than in-the-lab study) and the survey (EMA) respondent fatigue may have further impacted our results.

## Conclusions and future work areas

In this paper, we presented an attempt to model and predict smartphone application QoE from a living lab study, with 38 participants for four weeks. We showed that collecting in-situ QoE rating and collecting smartphone background data enables us to use common machine learning techniques to build an accurate predictive model for “High” and “Low” QoE. We investigated multiple data filtering scenarios that generated more accurate models in different scenarios. The data preparation (e.g., filtering and aggregation) allowed an improvement in our QoE models’ performance. The filtering of the participant QoE ratings was overall beneficial to the models. Namely, the models were performing better when trained on ratings provided closer to the application usage time. We investigated the factors influencing QoE in our dataset. Our results showed that rating the application usage session, just after the usage, permitted more reliable models. The task to accomplish with the application, by the user, and the application itself are important factors, testifying on the difficulty of generalization for this type of all-application QoE model, contrary to the per-app QoE model. We determined that application developers should have user expectations in mind when designing an application. We found expectation based QoE models to perform better. The mobile operating systems and their applications are more than ten-years-old. Their users now have a high expectation of how the application and the system will behave. We extended our work to the challenging domain of on-device prediction models, its difficulty, and its performance. Overall, our hybrid qualitative and quantitative method performed accurately to model QoE. In the future, we plan to implement a production-ready pre-trained prediction model integrating more features inside our Android application as the user’s position (on-device only). The application will predict if, in the near future (e.g., 5 minutes), the current QoE application usage session will be “High” or “Low”. If the prediction shifts because of the context (e.g., train inside a tunnel), the application can inform the user and prepare itself for the change. Those predictions, rated by the user, would allow us to use reinforcement learning to enhance our model comparable to recommendation systems. We also plan to integrate other factors influencing living lab study and potentially the collected data quality: the other aspects of the user’s context (e.g., mental state), previous experience, surroundings, operating system updates, and newly available features.

## Footnotes

1 *XBG*, *TR*, *FA*, and *ULT* models.
